# A technique for approximating transition rates from published survival analyses

**DOI:** 10.1186/s12962-019-0182-7

**Published:** 2019-07-01

**Authors:** Markian A. Pahuta, Joel Werier, Eugene K. Wai, Roy A. Patchell, Doug Coyle

**Affiliations:** 1 Department of Orthopaedic Surgery, Henry Ford Health System, Detroit, MI USA; 2 Division of Orthopaedic Surgery, The University of Ottawa, Ottawa, ON Canada; 3 School of Epidemiology and Public Health, The University of Ottawa, Ottawa, ON Canada; 4 Departments of Neurology and Neurosurgery, University of Kentucky Medical Center, Lexington, KY USA

**Keywords:** Cancer, Multistate model, Survival analysis, Quality adjusted life year

## Abstract

**Background:**

Quality-adjusted-life-years (QALYs) are used to concurrently quantify morbidity and mortality within a single parameter. For this reason, QALYs can facilitate the discussion of risks and benefits during patient counseling regarding treatment options. QALYs are often calculated using partitioned-survival modelling. Alternatively, QALYs can be calculated using more flexible and informative state-transition models populated with transition rates estimated using multistate modelling (MSM) techniques. Unfortunately the latter approach is considered not possible when only progression-free survival (PFS) and overall survival (OS) analyses are reported.

**Methods:**

We have developed a method that can be used to estimate *approximate transition rates* from published PFS and OS analyses (we will refer to transition rates estimated using full multistate methods as *true transition rates*).

**Results:**

The approximation method is more accurate for estimating the transition rates out of health than the transition rate out of illness. The method tends to under-estimate true transition rates as censoring increases.

**Conclusions:**

In this article we present the basis for and use of the transition rate approximation method. We then apply the method to a case study and evaluate the method in a simulation study.

**Electronic supplementary material:**

The online version of this article (10.1186/s12962-019-0182-7) contains supplementary material, which is available to authorized users.

## Background

**Fig. 1:**
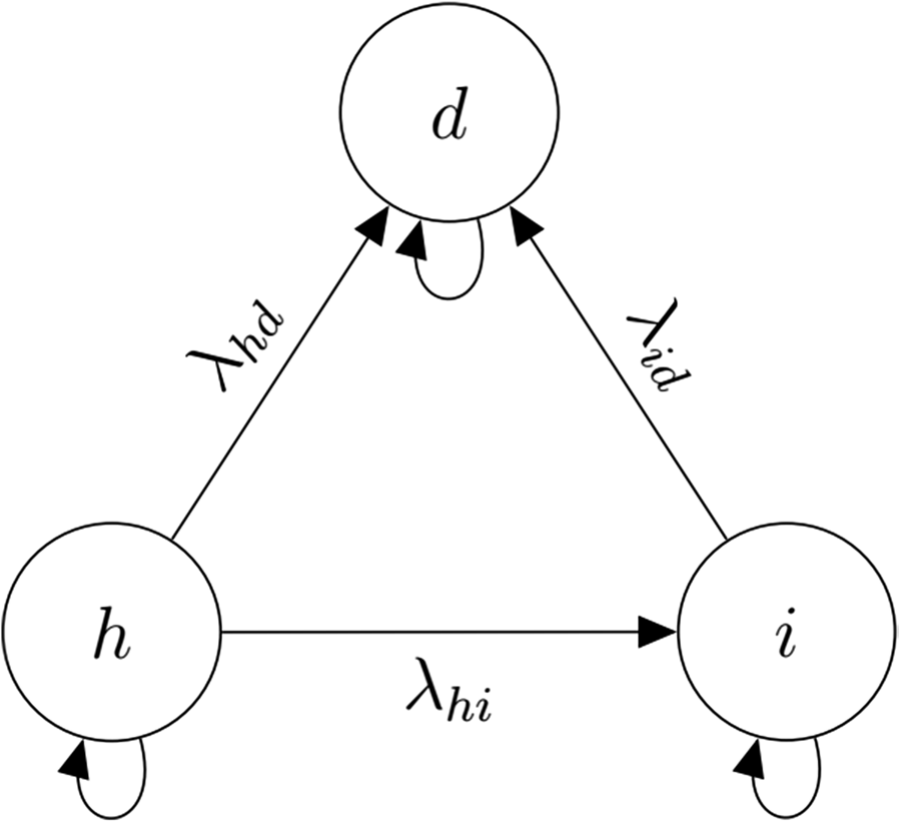
State-transition diagram for illness-death model. State-transition diagram for an illness-death model. The model consists of three health states: healthy (*h*), ill (*i*), and dead (*d*). Variable names adjacent to the solid arrows are transition rates ($\lambda _{hi}$, $\lambda _{hd}$, and $\lambda _{id}$). This model is said to be “progressive” because transitions are irreversible (i.e. unidirectional). The curved arrows indicate that individuals can remain in a particular state over time. See text for more details

Chronic, progressive, and non-communicable diseases (such as cancer, diabetes, cardiovascular disease and chronic respiratory disorders) are now the leading cause of morbidity and mortality around the world. More than 60% of global deaths are attributable to these types of diseases [1]; consequently these diseases now account for up to 50% of the total healthcare budget in some countries [2]. Many of these diseases can be conceptualized as consisting of three health states: healthy (*h*), ill (*i*), or dead (*d*) (Fig. 1).

Treatment decisions for chronic, progressive, and non-communicable diseases are difficult because interventions can have distinct, and sometimes opposite, influences on the probability that a patient experiences a given health state. For example, a therapy (e.g. high-risk cancer surgery) may decrease the risk of death (by controlling cancer) but may increase the risk of becoming ill (if a post-operative complication occurs). Quality-adjusted-life-years (QALYs) can be used to concurrently quantify morbidity and mortality within a single parameter [3]. For this reason, QALYs may facilitate the discussion of risks and benefits during patient counseling regarding treatment options [4]. QALY calculation requires knowledge of *state-membership fractions*. These are the proportion of patients from a defined cohort that are in a given health state at a given time *t*. State-membership fractions can be calculated using *partitioned-survival modelling* or *state-transition modelling* [5–7].

Partitioned-survival modelling uses data abstracted from progression-free survival (PFS) curves and overall survival (OS) curves reported in the literature [7]. PFS curves show the fraction of the cohort that is healthy over time *t* (*PFS*(*t*); OS curves show the fraction of the cohort that is alive (either healthy or ill) over time *t* (*OS*(*t*)). Since OS curves show the fraction of alive patients, the fraction of dead patients is simply $1-OS(t)$. The fraction of ill (but alive) patients is the difference between the fraction of alive and healthy patients $OS(t)-PFS(t)$. We will refer to state-membership fractions calculated in this way as *partitioned-survival fractions* [5–7]. In contrast, state-transition modelling applies the results of a *multistate analysis*. For the disease shown in Fig. 1, these techniques would be used to estimate the transition rate (i.e. the instantaneous risk (or hazard) of moving from one state to another) from health to illness ($h \rightarrow i$), from health to death ($h \rightarrow d$), and from illness to death ($i \rightarrow d$) [6]. Transition rates can be used compute transition probability matrices to calculate state-membership fractions (“multistate fractions”). It is important to recognize that state-transition modelling is based on a set of mutually exclusive health states (health, illness, death), whereas partitioned survival modelling is based on non-mutually exclusive health states (health and illness or death for the PFS curve, and alive and dead for the OS curve). Partitioned-survival modelling is used when sufficient data for state-transition modelling is unavailable.

QALY calculations based on partitioned-survival fractions can suffer from two important limitations that result from the fact that (i) the OS analysis does not consider the survival of ill patients separate from healthy patients, and (ii) the risk of progressing to illness rather than death for healthy patients cannot be determined from PFS analysis. The first limitation of partitioned-survival fractions stems from the difficultly of extrapolating partitioned-survival fractions beyond the study’s observation period [6]. This is a significant deficiency because clinical studies often have a limited observation period that is of insufficient duration to characterize long-term clinical outcomes [8–13]. The second limitation of partitioned-survival fractions is that computed QALYs are not generalizable to patient cohorts whose baseline fractions of healthy, ill and dead patients differs from those of the study cohort [6]. This is because the OS curve is a weighted average of OS curves for healthy and ill patients; therefore, the shape of the curve will change if the baseline ratio of healthy to ill patients differs. These two limitations restrict the use of partitioned survival fractions for decision analysis. These limitations can be avoided by calculating QALYs using multistate fractions. Because they are based on granular analyses of all transitions, multistate fractions have several advantages over partitioned-survival fractions. First, they can be reliably extrapolated beyond the study observation period [6]. Second, they can be used for decision analysis in cohorts with baseline characteristics that differ from the original study cohort [14].

Unfortunately, one cannot usually calculate transition rates using data abstracted from PFS and OS analyses [6, 7]. Given the limitations of partitioned-survival fractions and the advantages of multistate fractions, it would be helpful to obtain transition rates and calculate the latter when one only has access to PFS and OS analyses. We have developed a method that, under particular conditions, can be used to estimate *approximate transition rates* from published PFS and OS analyses (we will refer to transition rates estimated using full multistate methods as *true transition rates*).

This article organized as follows. We first present the basis for and use of the transition rate approximation method. A case study is then reported in which we apply transition rate approximation to data from a randomized controlled trial (RCT) of treatments for metastatic epidural spinal cord compression (MESCC). We then report a simulation study evaluating the accuracy of the approximation method. In the last section we summarize and discuss our findings.

## Methods

The approximation technique is restricted to three-state progressive, time-homogenous Markov disease processes such as the one shown in Fig. 1 [15]. *Progressive* means that transitions are irreversible (i.e. cannot return to health from illness). *Time-homogenous*, means that transition rates do not change over time. *Markov* means that transition rates do not depend on disease history; in other words, the probability that a patient transitions from state *x* to state *y* during a particular time period is independent of their previous health state.

The data needed to use the approximation technique can be abstracted from most articles reporting PFS and OS analyses. The number of patients experiencing an event and number of censored patients in both the PFS ($N_{pfs}^e$ and $N_{pfs}^c$) and OS ($N_{os}^e$ and $N_{os}^c$) analyses can be determined from the article text or patients-at-risk risk table. To obtain the remaining data points, PFS and OS KM curves need to be digitized. Digitized KM curves can then be used to reconstruct individual patient data using validated algorithms to determine the event times in the PFS and OS analyses [16]. The approximation technique requires that we make note of the maximum observation time (event or censoring) in the PFS and OS analyses ($\tau _{pfs}$ and $\tau _{os}$ respectively). The area under the PFS and OS curves ($AUC_{pfs}$ and $AUC_{os}$ respectively) are calculated by summing the area under each step of the KM curve.

We denote $h \rightarrow i$, $h \rightarrow d$, and $i \rightarrow d$ transition rates as $\lambda _{hi}$, $\lambda _{hd}$, and $\lambda _{id}$. For the time-homogenous disease processes (i.e. constant transition rates), exit times from the (i) healthy state (i.e. $h \rightarrow i$ or $h \rightarrow d$ transition) and (ii) ill state (i.e. $i \rightarrow d$ transition) are exponentially distributed. Furthermore, once a patient exits health, the probability that they make an $h \rightarrow d$ transition is

$$\begin{aligned} \rho =\frac{\lambda _{hd}}{\lambda _{hi}+\lambda _{hd}} \end{aligned}$$

We will refer to $\rho$ as the risk of death for healthy patients. As there are only two possible transitions out of health, the probability that a transition out of the health state is an $h \rightarrow i$ transition is $1-\rho$.

The mean time of exit from the healthy state (i.e. mean progression-free survival time) is a biased measure in the presence of right censoring [17]. Instead we calculate the restricted mean progression free-survival time (${\mathrm {RMPFST}}^{-\tau }$) which is interpreted as the mean progression-free survival time if observation is restricted to a truncation time $\tau$ [18]. Since the exit time from health is exponentially distributed, the ${\mathrm {RMPFST}}^{-\tau }$ can be calculated as

1 $$\begin{aligned} {\mathrm {RMPFST}}^{-\tau }=\frac{1-e^{-\left( \lambda _{hi}+ \lambda _{hd}\right) \tau }}{\lambda _{hi}+\lambda _{hd}}. \end{aligned}$$

By definition, the area under the PFS curve is equal to ${\mathrm {RMPFST}}^{-\tau }$ when $\tau$ is set to the maximum observation time in the PFS analysis, $\tau _{pfs}$ [19, 20]. Using Formula 1, we can then numerically solve for $\lambda _{hi}+\lambda _{hd}$ using standard algorithmic methods [21]. Simultaneous events in the PFS and OS analyses indicate $h \rightarrow d$ transitions. Therefore, we can approximate the risk of death for healthy patients as

2 $$\begin{aligned} \rho \approx \frac{N_{simul}}{N_{pfs}^e}. \end{aligned}$$

To approximate $\lambda _{id}$ we need to use information gathered from the OS analysis. It is more challenging to define an exact formula for the restricted mean overall survival time ($\mathrm {RMOST}^{-\tau }$) than form the ${\mathrm {RMPFST}}^{-\tau }$ because exit from the alive state (i.e. healthy or ill) is defined by a mixture of two exponential distributions: exit from health and exit from illness. However, if we know the death times $o_i^e$ and censoring times $o_j^c$ for a cohort of alive patients, $N_{os}^e$ who had an observed event, and $N_{os}^c$ who were right censored, we can approximate ${\mathrm {RMOST}}^{-\tau }$ truncated to $\tau _{os}$, ${\mathrm {RMOST}}^{-\tau _{os}}$, using inverse probability weighting [22]

3 $$\begin{aligned} {RMOST}^{-\tau _{os}} \approx \left( \left( \frac{N_{os}^e+N_{os}^c}{N_{os}^e}\right) \sum \limits _{i=1}^{N_{os}^e}o_i^e + \sum \limits _{j=1}^{N_{os}^c}o_j^c \right) \left( \frac{1}{N_{os}^e+N_{os}^c}\right) . \end{aligned}$$

Next, we determine the total person-time of observation in the OS analysis

4 $$\begin{aligned} E_{os}= \sum \limits _{i=1}^{N_{os}^e}o_i^e + \sum \limits _{j=1}^{N_{os}^c}o_j^c. \end{aligned}$$

If censoring times are not denoted on the OS curve, it is not possible to determine $o_j^c$. However, we can rearrange Formula 3 to yield

$$\begin{aligned} \sum \limits _{j=1}^{N_{os}^c}o_j^c \approx {\mathrm {RMOST}}^{-\tau _{os}} \left( N_{os}^e+N_{os}^c \right) - \sum \limits _{i=1}^{N_{os}^e}o_i^e\left( \frac{N_{os}^e+N_{os}^c}{N_{os}^e}\right) . \end{aligned}$$

If we substitute this relationship into Formula 4 we obtain

5 $$\begin{aligned} E_{os}&\approx \sum \limits _{i=1}^{N_{os}^e}o_i^e+ {\mathrm {RMOST}}^{-\tau _{os}} \left( N_{os}^e+N_{os}^c \right) -\sum \limits _{i=1}^{N_{os}^e}o_i^e \left( \frac{N_{os}^e+N_{os}^c}{N_{os}^e}\right) \nonumber \\&\approx {\mathrm {RMOST}}^{-\tau _{os}}\left( N_{os}^e+N_{os}^c \right) + \sum \limits _{i=1}^{N_{os}^e}o_i^e \left( 1- \frac{N_{os}^e+N_{os}^c}{N_{os}^e} \right) \end{aligned}$$

We can repeat the same calculations using the corresponding data from the PFS analysis to approximate total person-time of observation in the OS analysis, $E_{pfs}$. We then approximate the total person-time of observation in the ill state as

6 $$\begin{aligned} E_{ill} \approx E_{os} - E_{pfs}. \end{aligned}$$

If we make the assumption that the number of $i \rightarrow d$ transitions is

7 $$\begin{aligned} N_{id} \approx N_{os}^e - N_{simul}, \end{aligned}$$

we can compute [23]

8 $$\begin{aligned} \lambda _{id} \approx \frac{N_{id}}{E_{ill}}. \end{aligned}$$

## Results

### MESCC case study

To evaluate whether the approximation method can generate reasonable results, we compared approximate transitions rates against a gold standard of true transition rates estimated from real study data.

Patchell et al. [24] conducted a randomized controlled trial (RCT) comparing modern surgery and radiotherapy (mS+RT) versus radiotherapy alone (RT-alone) for the treatment of metastatic epidural spinal cord compression (MESCC). MESCC occurs when cancer metastasizes to the spine which and can lead to loss of ambulation from paralysis. MESCC can be modelled as in Fig. 1 if we consider ability to ambulate as the healthy state *h* and the inability to ambulate due to neurologic dysfunction as the ill state *i*. True transition rates were estimated using individual patient data provided by the study authors. To eliminate the potential for transcription error and inaccuracy in individual patient data reconstruction, we used actual individual patient data to generate the data listed in Table 1. We estimated true transition rates using the Bayesian modeling language Stan, [25] run through the statistical programming language R (Additional file 1: Appendix A) [26]. The effect of mS+RT was parametrized as a log-hazard ratio for each RT-alone transition rate.

**Table 1:** Data abstracted from MESCC RCT PFS and OS analyses

	Description	RT-alone arm	mS+RT arm
*PFS analysis*			
$N_{pfs}^e$	Total number of PFS events	17	31
$N_{pfs}^c$	Total number of patients censored from PFS analysis	5	6
$\sum \limits _{i=1}^{N_{pfs}^e}p_i^e$	Person-time of PFS observation	7.02 years	27.22 years
$\tau _{pfs}$	Maximum observation time in the PFS analysis	2.97 years	5.25 years
$AUC_{pfs}$	Area under PFS curve	0.63	1.16
*OS analysis*			
$N_{os}^e$	Total number of OS events	44	45
$N_{os}^c$	Total number of patients censored from OS analysis	1	3
$\tau _{os}$	Maximum observation time in the OS analysis	2.99 years	5.25 years
$\sum \limits _{i=1}^{N_{os}^e}o_i^e$	Person-time of OS observation	24.17 years	35.89 years
$AUC_{os}$	Area under OS curve	0.62	0.98
*Synthesis of PFS and OS analyses*			
$N_{simul}$	Total number of simultaneous events in PFS and OS curves	10	19

Prior to comparing true and approximate transition rates, we conducted non-parametric multistate analysis to assess whether our assumed model (progressive, time-homogenous and Markov) was appropriate for MESCC. Non-parametric multistate fractions were estimated from individual patient data from the MESCC RCT using the etm library [27] run through the statistical programming language R [26]. We compared non-parametric multistate fractions and multistate fractions calculated from true transition rates. Goodness-of-fit tests for true multistate analysis of data observed with exact transition times affected by right censoring have not been developed [5]. We therefore used informal graphical methods.

**Fig. 2:**
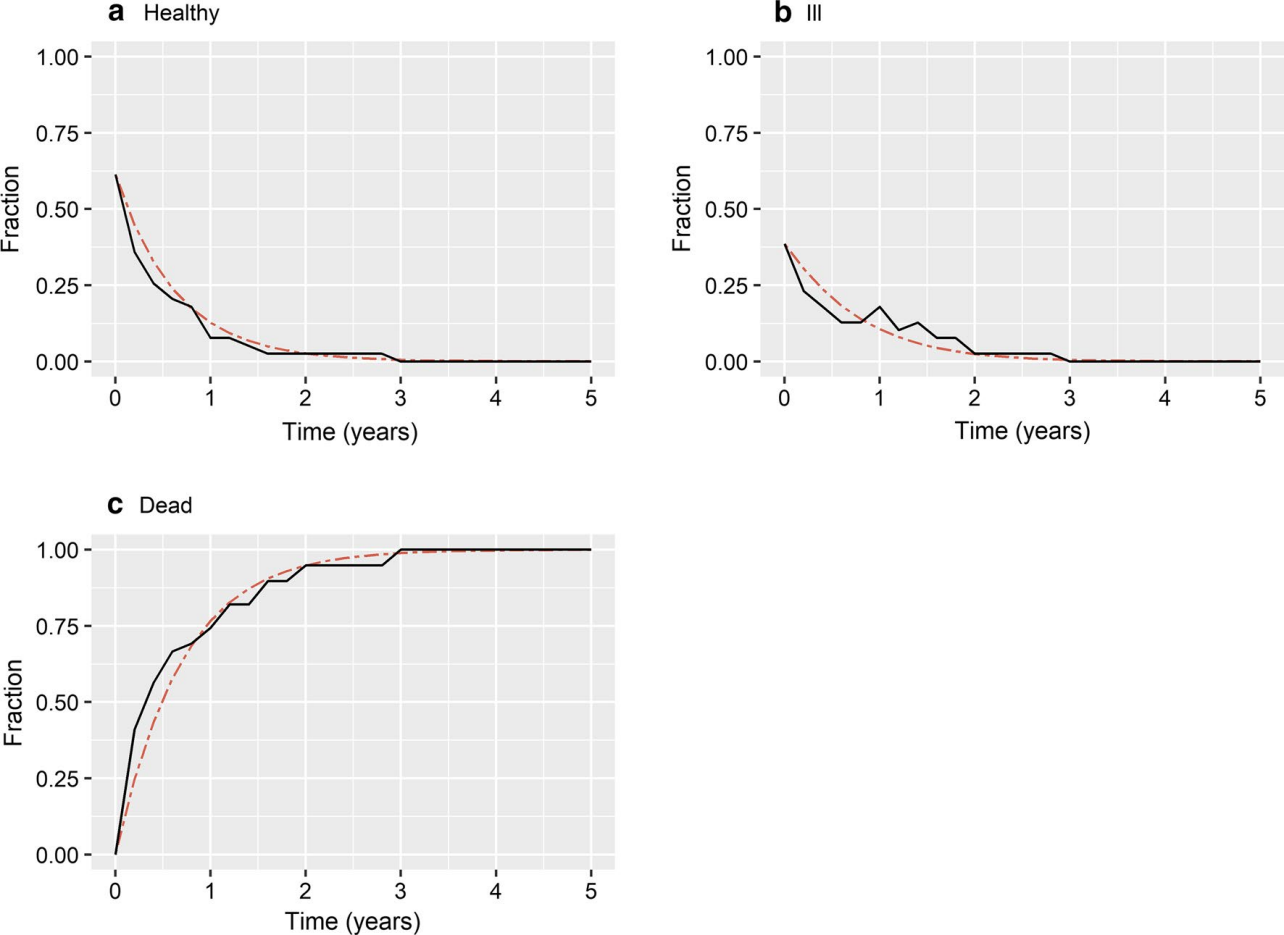
Comparison of state-membership fractions, RT-alone arm. State-membership fractions for RT-alone arm. Non-parametric multistate fractions, solid black line. Parametric multistate fractions based on true transition rates, small dashed red line

**Fig. 3:**
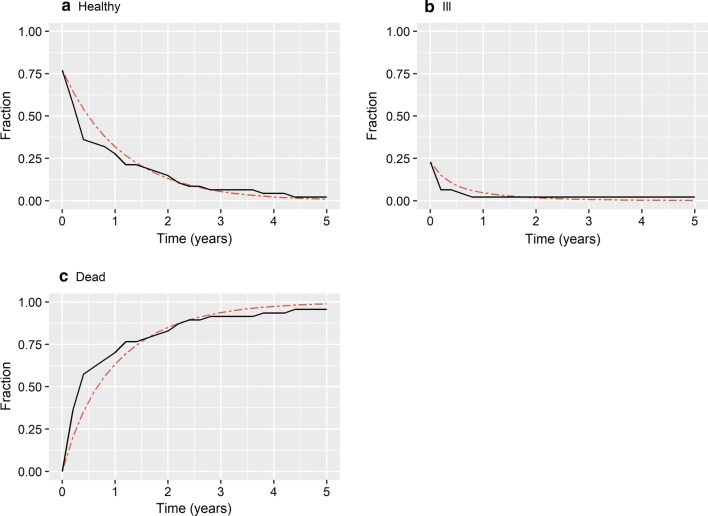
Comparison of state-membership fractions, mS+RT arm. State-membership fractions for arm. Non-parametric multistate fractions, solid black line. Parametric multistate fractions based on true transition rates, small dashed red line

Plots comparing proper non-parametric multistate and proper parametric multistate fractions showed good agreement, and no evidence of systematic deviation (Figs. 2 and 3). Therefore, a progressive time-homogenous three-state Markov model is appropriate for the MESCC RCT data and true transition rates can serve as an appropriate comparator to evaluate approximate transition rates. Calculations for the mS+RT arm are shown in Additional file 1: Appendix B.

The true transition rates shown in Table 2 provides useful insights into the impact of treatment. mS+RT prolongs ambulation with a statistically significant hazard ratio of 0.53 (95% CrI: 0.30, 0.94) on the total transition rate for exit from the ambulatory state $\left( \lambda _{hi}+\lambda _{hd}\right)$. For patients making a transition out of the ambulatory state, the risk of death was similar with both treatments: relative risk 1.07 (95% CrI, 0.65 – 1.75). mS+RT tended to increase the mortality rate for non-ambulatory patients, hazard ratio for $\lambda _{id}$ of 1.61 (95% CrI: 0.89, 2.66), but this effect was not statistically significant.

**Table 2:** Comparison of true and approximate transition rates and hazard ratios for MESCC RCT

	Transition rates				Hazard ratios			
	True (95% CrI)	Approximate	Error	% Error	True (95% CrI)	Approximate	Error	% Error
*RT-alone group*								
$\lambda _{hi}$	$0.66~ \left( 0.27,1.20\right)$	0.65	− 0.01	− 1.52				
$\lambda _{hd}$	$1.00~ \left( 0.50, 1.67\right)$	0.92	+ 0.08	− 8.00				
$\lambda _{id}$	$2.12~ \left( 1.40, 2.98\right)$	2.19	+ 0.07	+ 3.30				
mS+RT group								
$\lambda _{hi}$	$0.33~ \left( 0.17, 0.54\right)$	0.33	0.00	0.00	$0.50~ \left( 0.21, 1.40\right)$	0.51	+ 0.01	+ 1.54
$\lambda _{hd}$	$0.53~ \left( 0.32, 0.80\right)$	0.52	− 0.01	− 1.89	$0.55~ \left( 0.26, 1.16\right)$	0.56	+ 0.02	+ 2.77
$\lambda _{id}$	$3.28~ \left( 2.09, 4.69\right)$	3.71	+ 0.43	+ 13.11	$1.61~ \left( 0.89, 2.66\right)$	1.62	+ 0.08	+ 5.22

CrI, Bayesian credible interval. Error, Approximate − True. % Error, (Approximate − True) $\div$ True

All approximate transition rates lay within the 95% credible intervals for true transition rates. There was no consistent direction of error indicating the approximation method does not consistently under-or over-estimate true transition rates.

### Simulation study

To assess the validity of the approximation strategy in a wider set of conditions, we conducted a simulation study to assess the impact of censoring on the accuracy of the approximation method for $\left( \lambda _{hi}+\lambda _{hd}\right)$, $\rho$, and $\lambda _{id}$.

Data were generated randomly for a three-state progressive, time-homogenous Markov disease process with parameters similar to those for the mS+RT arm from the MESCC trial. A simulated cohort of 100 patients, 75 of which were healthy at baseline, was created with $\lambda _{hi}=0.33$, $\lambda _{hd}=0.53$, $\lambda _{id}=3.28$. Events times were independently censored using a uniform distribution to achieve all combinations of 0, 2, 5, and 10 patients censored from the OS and PFS analysis. 100 000 replications were generated for each set of simulation conditions.

We calculated the mean error (ME), mean absolute error (MAE), mean percentage error (%ME), and mean absolute percentage error (%MAE) for each set of simulation conditions (Tables 3, 4, and 5). ME and %ME are a measure of the direction of bias (systematic over- or underestimation). MAE and %MAE are a measure of the magnitude of error, regardless of direction.

**Table 3:** Simulation results for $\left( \lambda _{hi}+\lambda _{hd}\right)$

# Censored OS	# Censored PFS	ME	MAE	%ME	% MAE
0	0	0.00	0.08	0.11	9.34
5	8	− 0.06	0.10	− 7.33	11.19
5	15	− 0.10	0.12	− 12.18	14.09
5	30	− 0.21	0.21	− 24.69	24.86
10	15	− 0.10	0.12	− 11.50	13.70
10	30	− 0.20	0.21	− 23.69	23.93
20	15	− 0.08	0.11	− 9.85	12.74
20	30	− 0.19	0.19	− 22.32	22.65
40	30	− 0.17	0.17	− 19.30	20.01

**Table 4:** Simulation results for $\rho$

# Censored OS	# Censored PFS	ME	MAE	%ME	% MAE
0	0	− 0.00	0.04	− 0.01	7.27
5	8	− 0.00	0.05	− 0.66	7.90
5	15	− 0.00	0.05	− 0.62	8.24
5	30	− 0.00	0.06	− 0.58	9.50
10	15	− 0.01	0.05	− 1.42	8.30
10	30	− 0.01	0.06	− 1.33	9.52
20	15	− 0.02	0.05	− 3.75	8.70
20	30	− 0.02	0.06	− 3.05	9.74
40	30	− 0.06	0.08	− 9.38	12.21

**Table 5:** Simulation results for $\lambda _{id}$

# Censored OS	# Censored PFS	ME	MAE	%ME	% MAE
0	0	0.07	0.41	2.25	12.42
5	8	− 0.41	0.56	− 12.59	17.19
5	15	− 0.55	0.72	− 16.81	21.92
5	30	− 69.35	71.28	− 2114.42	2173.07
10	15	− 0.52	0.70	− 15.96	21.35
10	30	− 0.73	2.36	− 22.13	72.08
20	15	− 4.54	4.60	− 138.32	140.30
20	30	0.79	2.75	24.22	83.88
40	30	− 1.89	1.90	− 57.67	57.88

The approximation method tended to underestimate $\left( \lambda _{hi}+\lambda _{hd}\right)$ and $\rho$ as the censoring rate increased, however the bias was small with %ME under 3% in all censoring conditions. Even under no censoring, the approximation method was imprecise with a relatively high MAE and %MAE; increasing censoring did not significantly decrease precision.

The approximation method tended to underestimate $\lambda _{id}$ as the censoring rate increased, however the bias was small with %ME under 3% in all censoring conditions. Even under no censoring, the approximation method was imprecise with a relatively high MAE and %MAE; increasing censoring did not significantly decrease precision.

The approximation method tended to underestimate $\lambda _{id}$ as the censoring rate increased, however the bias was small with %ME under 3% in all censoring conditions. Even under no censoring, the approximation method was imprecise with a relatively high MAE and %MAE; increasing censoring did not significantly decrease precision.

## Discussion

Although chronic, progressive, and non-communicable diseases chronic diseases affect both patients’ survival and quality-of-life, interventions may impact on these two outcomes differentially. QALYs can simplify decision-making and counselling regarding treatment options [4]. For clinicians and decision makers, QALYs calculated using multistate fractions are useful because they can be used to extrapolate long-term quality-of-life and to conduct rich decision analysis. Unfortunately, one cannot usually calculate multistate fractions from PFS and OS curves [5–7].

In this paper, we presented a technique for approximating transition rates, which can be used to calculate multistate fractions, from PFS and OS analysis. Our technique requires that three elements be abstracted from each of the PFS and OS analyses: (i) total number of events, (ii) total number of censored patients, and (iii) event times.

Approximate transition rates provide a reasonable estimate of true transition rates estimated using full multistate methods. For the MESCC RCT case study, all approximate transition rates lay within the 95% Bayesian credible intervals for true transition rates. The simulation study indicates that the approximation method is relatively unbiased and precise for estimating the transition rate out of health $\left( \lambda _{hi}+\lambda _{hd}\right)$ and the risk of death for healthy patients $\rho$.

It is important to recognize that our techniques only apply to a time-homogenous progressive three-state irreversible disease process. Time-homogeneity is violated if the transition rates change with time (i.e. any parametric model aside from the exponential) or depend on the amount of time spent in the preceding health state (non-Markov phenomenon) [28]. Irreversibility is violated if patients can become healthy after being ill [15]. Our approximation approach can be scaled-up to more complex (e.g. reversible transitions, > 3 health states) disease models, however, the formulas will become more complex. Furthermore, as was done in this article, it would be necessary to validate the scaled-up approximation approach to evaluate for bias.

## Conclusions

In this paper, we have demonstrated that transition rates can be approximated from published PFS and OS analyses. The approximation method is more accurate for estimating the transition rates out of health than the transition rate out of illness. The method tends to under-estimate true transition rates as censoring increases; therefore, approximate transition rates are not a substitute for true transition rates estimated with full multistate methods. However, when proper multistate analysis is not available, approximate transition rates can guide probabilistic modeling and enhance QALY analysis if one considers and accounts for the limitations of the approximation method.

## Additional files

**Additional file 1: Appendix A.** Transition parameters for an illness-death model.** Appendix B.** Multistate estimation of transition rates.

## Data Availability

The datasets used and/or analysed during the current study are available from the corresponding author on reasonable request.

## Abbreviations

MAE: mean absolute
%MAE: mean absolute percentage error
ME: mean error
%ME: mean percentage error
MESCC: metastatic epidural spinal cord compression
mS+RT: modern surgery followed by radiotherapy
OS: overall survival
PFS: progression-free survival
QALY: quality-adjusted life-years
RCT: randomized controlled trial
RT-alone: radiotherapy alone
