# Supplementary material for: A technique for approximating transition rates from published survival analyses
Source: Cost Eff Resour Alloc. 2019 Jul 1;17:12. doi: 10.1186/s12962-019-0182-7 (PMC6604134; doi:10.1186/s12962-019-0182-7)
Supplement: Supplementary file 1 — Additional file 1: Appendix A. Transition parameters for an illness-death model. Appendix B. Multistate estimation of transition rates. [file 12962_2019_182_MOESM1_ESM.pdf]

Appendix A

Transition Parameters for an Illness-Death Model

The transition rates between states can be organized in a transition rate matrix $\mathbf{R}$ where rows correspond to the "from" state, and the columns to the "to" state. States in the illness-death model are healthy (h), ill (i), and dead (d). Disallowed transitions are given a value of 0, and all rows must sum to 0. We model the MESCC disease process using time-homogenous transition rates: that is transition rates do not change over time and thus $\mathbf{R}$ is constant over time. Disallowed transitions are i to h, and d to either h or i. The transition rate matrix is

$$\begin{bmatrix} -\left(\lambda_{hi}+\lambda_{hd}\right) & \lambda_{hi} & \lambda_{hd} \\ 0 & -\lambda_{id} & \lambda_{id} \\ 0 & 0 & 0 \end{bmatrix}.[3]$$

The transition rate matrix can be converted to a transition probability matrix $\mathbf{P}$. This matrix records the probability of making transitions in the time interval from $t = 0$ to the time t. Using the transition rate matrix $\mathbf{R}$, the transition probability matrix is computed as

$$\begin{aligned} \mathbf{P}\left(t\right) &= e^{\mathbf{R}t} \\ &= \begin{bmatrix} p_{hh}\left(t\right) & p_{hi}\left(t\right) & p_{hd}\left(t\right) \\ 0 & p_{id}\left(t\right) & p_{id}\left(t\right) \\ 0 & 0 & 0 \end{bmatrix} \\ &= \begin{bmatrix} e^{-\left(\lambda_{hi}+\lambda_{hd}\right)t} & -\frac{\lambda_{an}e^{-\lambda_{id}t}\left(-1+e^{-\left(\lambda_{hi}+\lambda_{hd}-\lambda_{id}\right)t}\right)}{\lambda_{hi}+\lambda_{hd}-\lambda_{id}} & 1-p_{hh}\left(t\right)-p_{hi}\left(t\right) \\ 0 & e^{-\lambda_{id}t} & 1-p_{ii}\left(t\right) \\ 0 & 0 & 0 \end{bmatrix}.[3] \end{aligned}$$

The fraction of patients in each state at time t is recorded in the state membership vector $\mathbf{m}\left(t\right)$. The initial state membership fractions are $\mathbf{m}\left(0\right) = \left[\theta_h, \theta_i, \theta_d\right]$. $\mathbf{m}\left(t\right)$ is computed as the product of $\mathbf{m}\left(0\right)$ and the transition probability matrix $\mathbf{P}\left(t\right)$,

$$\mathbf{m}\left(t\right) = \mathbf{P}\left(0\right) \cdot \mathbf{P}\left(t\right).$$

The first element of $\mathbf{m}(t)$ is $H(t)$, the fraction of healthy patients

$$H(t) = \theta_h e^{-(\lambda_{hi}+\lambda_{hd})t}.$$

The second element of $\mathbf{m}(t)$ is $I(t)$, the fraction of ill patients

$$I(t) = \theta_i e^{-\lambda_{id}t} - \frac{\theta_h \lambda_{hi} e^{-\lambda_{id}t}\left(-1 + e^{-(\lambda_{hi}+\lambda_{hd}-\lambda_{id})t}\right)}{\lambda_{hi}+\lambda_{hd}-\lambda_{id}}.$$

The third element of $\mathbf{m}(t)$ is $D(t)$, the fraction of dead patients

$$D(t) = 1 - \theta_h e^{-(\lambda_{hi}+\lambda_{hd})t} - \theta_i e^{-\lambda_{id}t} + \frac{\theta_h \lambda_{hi} e^{-\lambda_{id}t}\left(-1 + e^{-(\lambda_{hi}+\lambda_{hd}-\lambda_{id})t}\right)}{\lambda_{hi}+\lambda_{hd}-\lambda_{id}}.$$

Appendix B

Multistate Estimation of Transition Rates

Methods

Likelihood

From Patchell *et al.*'s trial data, patients' health state trajectories can be categorized into seven different sequences (Table B.1). For some patients, the state was unknown at some time intervals. The time spent in the h, i and unknown state are denoted t_h, t_i, and t_u respectively. The transition rate and transition probability matrices are used to calculate the likelihood contribution for each trajectory.[8, 7, 5]

Table B.1: Health state trajectories and corresponding likelihood contributions

Sequence of states	Likelihood contribution
$h \to i \to d$	$\lambda_{hi} p_{hh}(t_h) \times \lambda_{id} p_{ii}(t_i)$
$h \to d$	$\lambda_{hd} p_{hh}(t_h)$
$i \to d$	$\lambda_{id} p_{ii}(t_i)$
$h \to$ censored	$p_{hh}(t_h)$
$i \to$ censored	$p_{ii}(t_i)$
$h \to$ unknown $\to d$	$p_{hh}(t_h) \times (\lambda_{hd} p_{hh}(t_u) + \lambda_{id} p_{ii}(t_u) + \lambda_{id} p_{hi}(t_u))$
unknown $\to d$	$\lambda_{hd} p_{hh}(t_u) + \lambda_{id} p_{ii}(t_u) + \lambda_{id} p_{hi}(t_u)$

Prior Distributions

Non-informative prior distributions were used for multistate model estimation

$$\begin{aligned} \log\lambda &\sim \text{Normal}\left(\text{mean}=0,\text{variance}=10\right), \\ \beta &\sim \text{Normal}\left(\text{mean}=0,\text{variance}=10\right). \end{aligned}$$ [9]

An upper truncation limit of 10 was applied to all prior distributions.[9]

Implementation

The model was estimated using the Bayesian modelling language Stan[12] run through the statistical programming language R.[10] Four Markov chains were implemented with different random initial values for each chain.

Initially all chains were run for 5000 iterations without thinning; 2500 burn-in iterations were discarded. Post-burn-in traceplots for each parameter were examined to ensure that all four chains reached a similar mean and sampled similar regions of the distribution.[6] Iterations were increased until (1) the potential scale reduction factor, $\hat{R}$, was < 1.1; and (2) histograms of the posterior distribution were smooth and without gaps.[2, 1, 4] To mitigate autocorrelation, thinning was increased so that effective sample size, N_{eff},was similar for all parameters, and the effective sample size as $\geq 20\%$ of the number of sampling iterations.[6] Post-burn in iterations were increased until Monte Carlo (MC) error for each parameter was $< 5\%$ of the sample standard deviation.[11] The final simulation conditions were termed the short conditions.

To ensure that global convergence was achieved (rather than local convergence), models was re-run with iterations doubled.[4] Simulation conditions were adjusted to ensure convergence and mitigate autocorrelation. These simulation conditions were termed the long conditions. Percent relative deviation for the estimates from the long conditions to the short conditions was computed. Global convergence was indicated if the percent relative deviation was less than $\pm 2\%$ for each parameter.[4]

STAN Code

```
functions{
  real MarkovKnownlpdf(real t, real [] lam, real censind, int state, int trans){
    //log-likelihood function when state known
    real lamstay [3]; real LL;
    lamstay[1]=-(lam[1]+lam[2]);
    lamstay[2]=-lam[3];
    LL = (lamstay[state])*t + log(lam[trans])*censind;
    return LL;
  }
  real MarkovUnknownlpdf(real t, real [] lam){
    //log-likelihood function when state unknown
real LL; real LikeA; real LikeB; real LikeC;
LikeA=exp((-lam[1] - lam[2])*t)*lam[2];
LikeB=(lam[1]/(lam[1]+lam[2]-lam[3]))*(exp(-lam[3]*t)-exp((-lam[1] - lam[2])*t))*lam[3];
LikeC=exp(-lam[3]*t)*lam[3];
LL = log(LikeA + LikeB +LikeC);
    return LL;
  }
}
data {
  int <lower=0> Nknown; int <lower=0> Nunknown; vector [Nknown] tknown; vector [Nunknown] tunknown;
  int <lower=0> stateknown [Nknown]; int <lower=0> transknown [Nknown]; vector [Nknown] censindknown;
  vector [Nknown] grpknown; vector [Nunknown] grpunknown;
}
parameters {
  real llam [3]; real bet [3];
}
transformed parameters {
  real lam [3]; real slam [3];
   for (k in 1:3){
    //transition rates for RT-alone group
      lam[k]=exp(llam[k]);
    //transition rates for mS+RT group
      slam[k]=lam[k]*exp(bet[k]);
  }
}
model {
  real tempknown [3]; real tempunknown [3];
  //likelihood for transitions where from and to state known
    for (i in 1:Nknown){
      tempknown[1]=lam[1]*exp(bet[1]*grpknown[i]);
      tempknown[2]=lam[2]*exp(bet[2]*grpknown[i]);
      tempknown[3]=lam[3]*exp(bet[3]*grpknown[i]);
      target += MarkovKnownlpdf(tknown[i], tempknown, censindknown[i], stateknown[i], transknown[i]);
    }
  //likelihood for transitions where either from or to state unknown
    for (j in 1:Nunknown){
      tempunknown[1]=lam[1]*exp(bet[1]*grpunknown[j]);
      tempunknown[2]=lam[2]*exp(bet[2]*grpunknown[j]);
      tempunknown[3]=lam[3]*exp(bet[3]*grpunknown[j]);
      target += MarkovUnknownlpdf(tunknown[j], tempunknown);
    }
  //Prior Distributions
    llam ~ normal(0, 10)T[,10];
    bet ~ normal(0, 10)T[,10];
}
```

Results

To meet pre-specified benchmarks for autocorrelation, thinning was increased to 10 to ensure that effective sample size as $\geq 20\%$ of the number of sampling iterations (Table B.2).

Traceplots for λ, and β showed a stable mean and variance under both short (Figure B.1)and long (Figure B.2) conditions. $\hat{R}$ was < 1.1 for all parameters under all conditions; and percent relative deviation was $< \pm 2\%$ for all parameters (Table B.2). We conclude that global convergence was reached.

Histograms of the posterior distribution under both short (Figure B.3) and long (Figure B.4) simulation conditions show smooth histograms without empty bins. Under both short and long simulation conditions, Monte Carlo error was $< 5\%$ of the sample standard deviation for each parameter (Table B.2). We conclude that both the short and long simulation conditions were sufficient to characterize the shape of the posterior distribution.

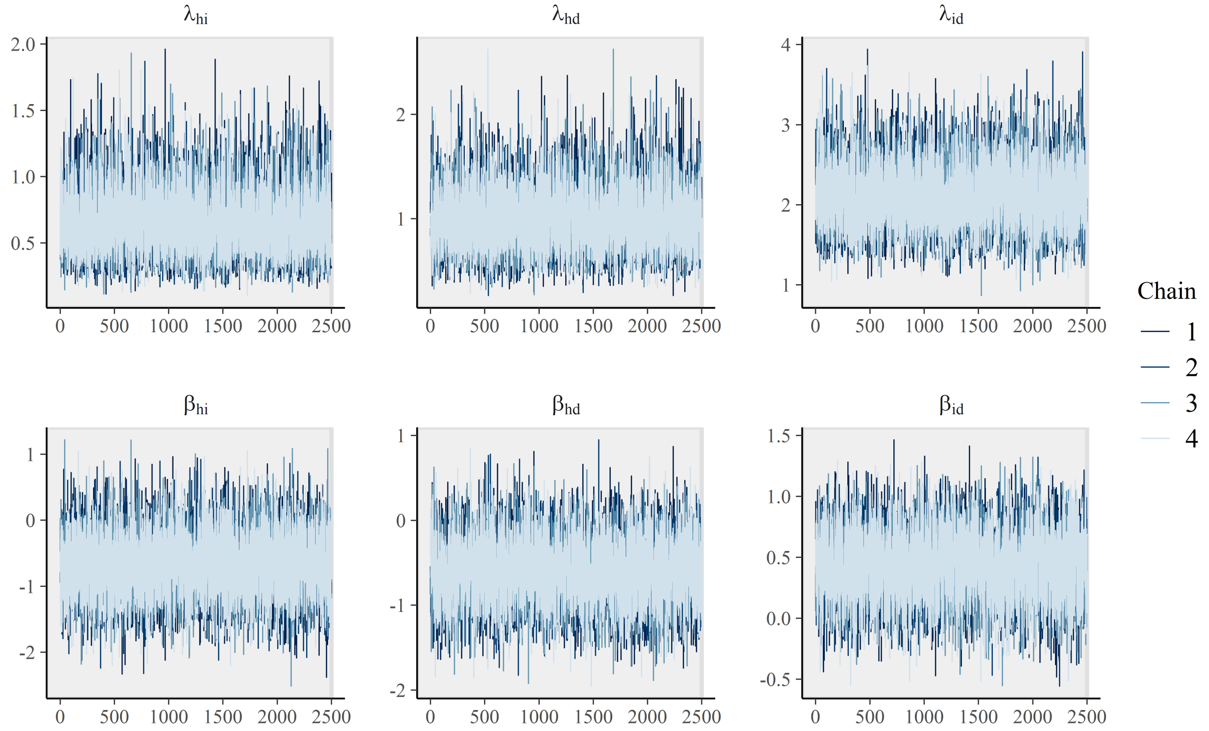

Figure B.1: Post-burn-in traceplots for transition rates and hazard ratios under short simulation conditions.

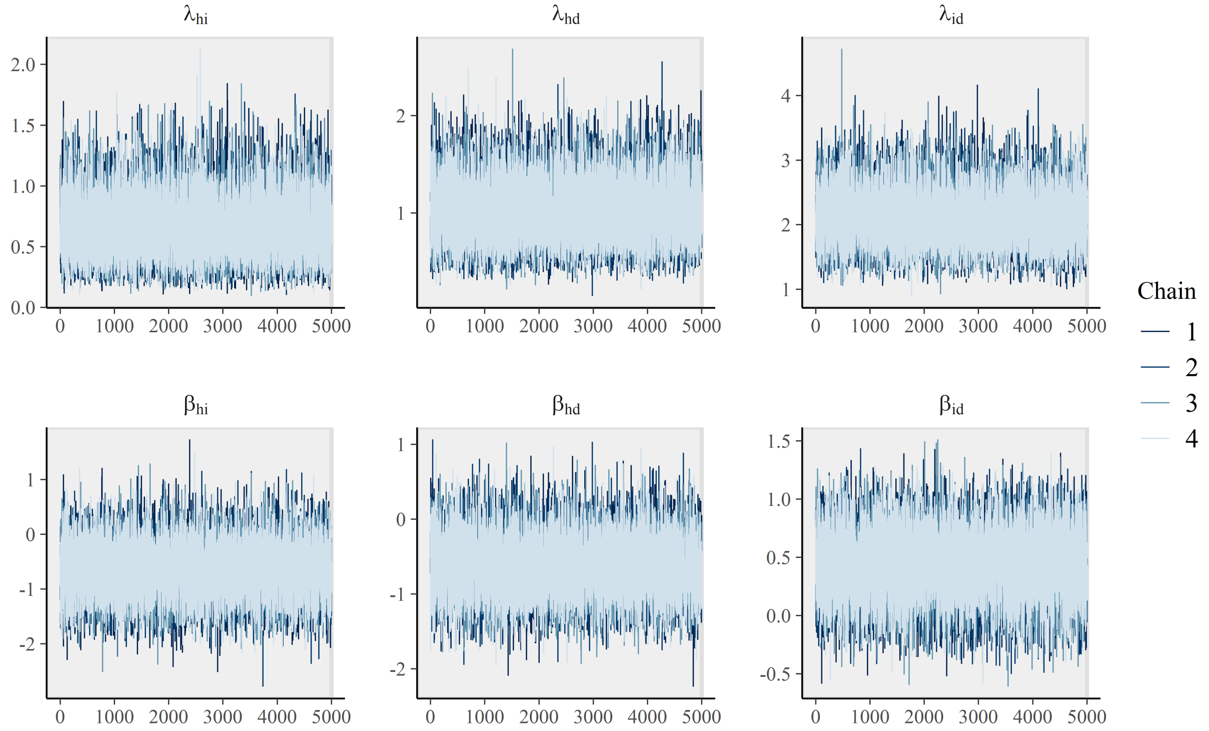

Figure B.2: Post-burn-in traceplots for transition rates and hazard ratios under long simulation conditions.

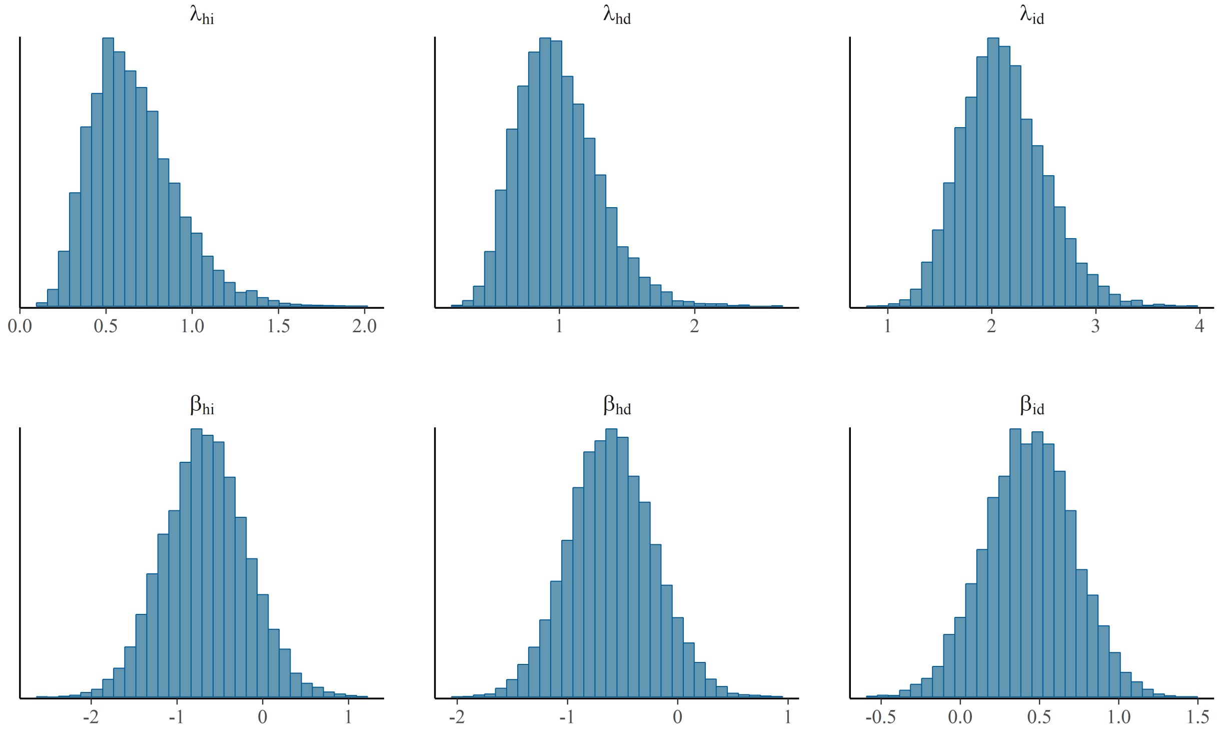

Figure B.3: Histograms of posterior distributions for transition rates and hazard ratios under short simulation conditions.

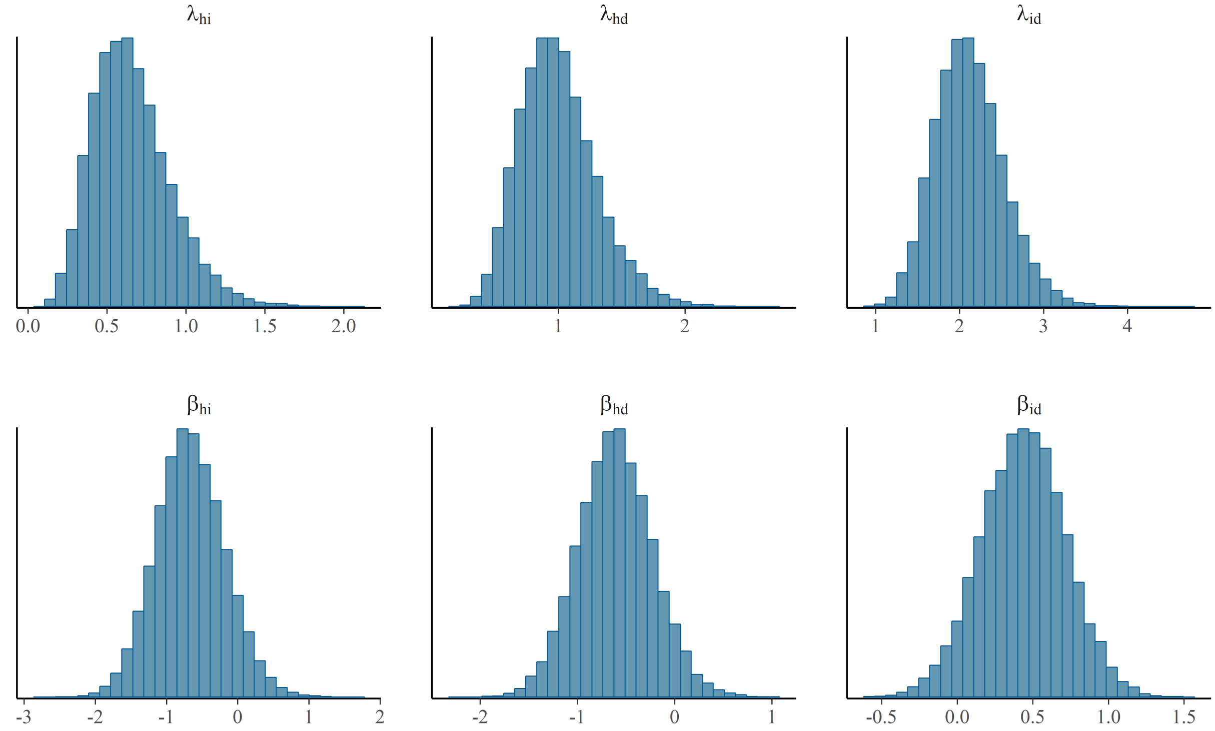

Figure B.4: Histograms of posterior distributions for transition rates and hazard ratios under short simulation conditions.

Table B.2: Comparison of Transition Rates and Hazard Ratios Estimated with Multistate Techniques under Short and Long Simulation Conditions

	Short Conditions					Long Conditions					% Rel
	Mean	SDev	MCSE	$\hat{R}$	N_{eff}	Mean	SDev	MCSE	$\hat{R}$	N_{eff}	Dev
λ_{hi}	0.66	0.25	0.00	10094	1.00	0.66	0.24	0.00	19751	1.00	0.80
λ_{hd}	0.99	0.30	0.00	9956	1.00	1.00	0.30	0.00	19084	1.00	-0.50
λ_{id}	2.12	0.40	0.00	10039	1.00	2.12	0.40	0.00	19627	1.00	0.00
β_{hi}	-0.66	0.49	0.00	10303	1.00	-0.66	0.48	0.00	19906	1.00	0.30
β_{hd}	-0.60	0.39	0.00	9665	1.00	-0.61	0.38	0.00	19387	1.00	-1.00
β_{id}	0.44	0.28	0.00	9805	1.00	0.44	0.28	0.00	19750	1.00	0.00

SDev, standard deviation. MCSE, Monte Carlo standard error. % Rel. Dev., percent relative deviation

References

[1] Stephen P Brooks and Andrew Gelman. General methods for monitoring convergence of iterative simulations. *J. Comput. Graph. Stat.*, 7(4):434–455, dec 2013.

[2] Mary Kathryn Cowles and Bradley P Carlin. Markov Chain Monte Carlo Convergence Diagnostics : A Comparative Review. *J. Am. Stat. Assoc.*, 91(434):883–904, 1996.

[3] David Roxbee Cox and Hilton David Miller. *The theory of stochastic processes*. Wiley, New York, 1965.

[4] Sarah Depaoli and Rens van de Schoot. Improving transparency and replication in Bayesian statistics: The WAMBS-Checklist. *Psychol. Methods*, 22(2):240–261, 2017.

[5] Paul David Feigin. Maximum likelihood estimation for continuous-time stochastic processes. *Adv. Appl. Probab.*, 8(04):712–736, dec 1976.

[6] Andrew Gelman, Bradley P. Carlin, Hal S. Stern, and Donald B. Rubin. *Bayesian Data Analysis*. Chapman and Hall/CRC, Boca Raton, second edition, 2003.

[7] J. D. Kalbfleisch and J. F. Lawless. The Analysis of Panel Data under a Markov Assumption. *J. Am. Stat. Assoc.*, 80(392):863–871, dec 1985.

[8] R Kay. A Markov model for analysing cancer markers and disease states in survival studies. *Biometrics*, 42(4):855–65, dec 1986.

[9] Malcolm J Price, Nicky J Welton, and A E Ades. Parameterization of treatment effects for meta-analysis in multi-state Markov models. *Stat. Med.*, 30(2):140–51, jan 2011.

[10] R Core Team. R: A Language and Environment for Statistical Computing, 2018.

[11] David J. Spiegelhalter, Andrew Thomas, Nicky Best, and David Lunn. WinBUGS User Manual: version 1.4. Technical report, MRC Biostatistics Unit, Cambridge, UK, 2003.

[12] Stan Development Team. The Stan Core Library, Version 2.17.0, 2017.
